# Durable complete response in a patient with metastatic melanoma following adoptive transfer of autologous T cells recognizing 10 mutated tumor antigens

**DOI:** 10.1186/2051-1426-3-S2-O4

**Published:** 2015-11-04

**Authors:** Todd D Prickett, Jessica S Crystal, Jared J Gartner, Yao Xin, Pasetto Anna, Alena Gros, Yong-Chen Lu, Yong Li, Mona El-Gamil, Steven A Rosenberg, Paul Robbins

**Affiliations:** 1 National Cancer Institute, National Institutes of Health, Bethesda, MD, USA; 2 National Cancer Institute, National Institutes of Health, Rutgers Robert Wood Johnson Medical School, New Brunswick, NJ, USA; 3 Surgery Branch, National Cancer Institute, National Institutes of Health, Bethesda, MD, USA

## 

Durable complete response rates of 20% have been observed in clinical trials of patients with metastatic melanoma employing adoptive cell transfer (ACT) of patient derived tumor infiltrating lymphocytes (TIL). Here we provide a detailed analysis of the response of TIL administered to a patient with metastatic melanoma who exhibited a complete response ongoing greater than 3 years. Using whole exome and RNA sequencing and bioinformatic analysis of the patient's matched tumor and normal gDNA we identified over 4,000 non-synonymous somatic mutation variants. We screened 745 somatically mutated genes using tandem minigene constructs expressing transcripts expressed in autologous tumor cells whose expression levels were greater than 0.1% of the levels of β- actin. These tandem minigenes were then transfected into autologous B cells and then analyzed for their ability to stimulate the administered T cells. Our results indicated that the autologous TIL distinctly recognized 10 somatically mutated gene products, each of which was recognized in the context of three different HLA class I restriction elements expressed by the patient's tumor. Detailed T cell clonal analysis revealed that 9 of the top 20 most prevalent clones present in the infused TIL, comprised over ¼ of total infused cells and recognized mutated antigens. These results further supported our efforts to identify and enrich mutation-reactive T cells for the treatment of patients with metastatic cancer.

